# Spiritual and Religious Journeys Over the Lifecourse: Challenging Stereotypes

**DOI:** 10.1093/geroni/igaa057.2360

**Published:** 2020-12-16

**Authors:** Keith Anderson, Cynthia Garthwait, Laurel Vielle

**Affiliations:** 1 University of Texas at Arlington, Arlington, Texas, United States; 2 University of Montana, Missoula, Montana, United States

## Abstract

Spiritual and religious beliefs often evolve across the lifecourse and tend to be influenced by experience, time, and maturity. Despite this evolution, stereotypes persist of older adults as being inflexible in their views and resistant to new or alternative beliefs. To explore this notion, researchers surveyed a convenience sample of 152 older adults (n = 152) who identified as spiritual/religious. Over three-quarters (77.0%) reported that their relationship with God or a higher power had grown closer over time. Most reported becoming “more liberal” (32.2%), “more conservative” (23.0%), or “more moderate” (17.8%) in religious perspective over time. Almost three-quarters of participants (73.7%) reported that they were “more accepting” of other religions”, yet also “more certain” of their own beliefs (74.3%) in later life. These and other results challenge the stereotype of older adults as “set in their ways”, even in terms of spiritual and religious beliefs.

